# Supplementary material for: Molecular epidemiology, genetic diversity and antimicrobial resistance of Staphylococcus aureus isolated from chicken and pig carcasses, and carcass handlers
Source: PLoS One. 2020 May 14;15(5):e0232913. doi: 10.1371/journal.pone.0232913 (PMC7224487; doi:10.1371/journal.pone.0232913)
Supplement: Attachment — Submitted filename: Response to Reviewers.docx [file pone.0232913.s001.docx]

1. Is the manuscript technically sound, and do the data support the conclusions?

The manuscript must describe a technically sound piece of scientific research with data that supports the conclusions. Experiments must have been conducted rigorously, with appropriate controls, replication, and sample sizes. The conclusions must be drawn appropriately based on the data presented.

Reviewer #1: Partly

Reviewer #2: Partly

2. Has the statistical analysis been performed appropriately and rigorously?

Reviewer #1: No

Reviewer #2: Yes

3. Have the authors made all data underlying the findings in their manuscript fully available?

The [PLOS Data policy](https://nam03.safelinks.protection.outlook.com/?url=http%3A%2F%2Fwww.plosone.org%2Fstatic%2Fpolicies.action%23sharing&data=02%7C01%7CTsmit176%40kent.edu%7C4398dc5b81cd467051c308d7b5272506%7Ce5a06f4a1ec44d018f73e7dd15f26134%7C1%7C0%7C637177053105469812&sdata=MPAW2Rkzzp1High6OBSgCtIBVgLi%2BMCLE7dg3qtlIwY%3D&reserved=0) requires authors to make all data underlying the findings described in their manuscript fully available without restriction, with rare exception (please refer to the Data Availability Statement in the manuscript PDF file). The data should be provided as part of the manuscript or its supporting information, or deposited to a public repository. For example, in addition to summary statistics, the data points behind means, medians and variance measures should be available. If there are restrictions on publicly sharing data—e.g. participant privacy or use of data from a third party—those must be specified.

Reviewer #1: Yes

Reviewer #2: Yes

4. Is the manuscript presented in an intelligible fashion and written in standard English?

PLOS ONE does not copyedit accepted manuscripts, so the language in submitted articles must be clear, correct, and unambiguous. Any typographical or grammatical errors should be corrected at revision, so please note any specific errors here.

Reviewer #1: No

Reviewer #2: Yes

5. Review Comments to the Author

Please use the space provided to explain your answers to the questions above. You may also include additional comments for the author, including concerns about dual publication, research ethics, or publication ethics. (Please upload your review as an attachment if it exceeds 20,000 characters)

Reviewer #1: Comments:
1. There are sloppy mistakes in the abstract including not abbreviating Staphylococcus after the first use, even the capitalization in the title is inconsistent.

**Response: This was changed to all lowercase and the abstract was edited.**

2. Inconsistent tense in lines 97-98

**Response: This has been edited.**

3. Line 162 they use t045, t002, and CC5 without any prior explanation. they need to write for a broader audience that doesn’t understand their jargon.

**Response: This has been edited.**

4. Line 187-190: I don’t think you can use Chi-square on percentages. There are much more applicable tests.

**Response: We did not use Chi-square on percentages. It was an error on our part. We have edited the statement**

5. Line 194 don’t repeat numbers in the text that are already in Table 1.

**Response: We removed all but the overall prevalence and significant difference between handlers and instructed readers to see Table** 1.

6. Table 1: add a more explanatory description of the contents, including what is CI. How is chicken 28/600 = 0.93%. I don’t need a calculator to know that is less than 0.5%. Same for 17 out of 600 for pig

**Response: These were typographical errors and have been corrected. We included the term confidence interval to indicate CI is an abbreviation but did not define it as we expect readers will be familiar with the term.**

7. Line 201: presence of the MecA doesn’t necessarily mean resistant to methicillin.

**Response: By the definition of methicillin-resistant *Staphylococcus aureus* (MRSA) strains, they are *Staphylococcus aureus* (*S. aurues*) strains that have an oxacillin MIC of ≥4µg/ml or harbor the *mecA* gene (Kumar et al., 2013). Chambers et al. (1997) also stated that methicillin resistance in Staphylococci is determined by *mecA* gene and that there is no *mecA* homolog in a susceptible strain of *S. aureus*, and so methodologies based on the detection of *mecA* gene are the most accurate.**

**However, *S. aureus* isolates that carry the *mecA* gene but appear phenotypically methicillin/ oxacillin susceptible have been increasingly reported (Ikonomidis et al., 2008) and so it has been suggested that such isolates be classified as a new type of MRSA, designated oxacillin-susceptible methicillin-resistant *S. aureus* (OS-MRSA).**

**Hence, Ikonomidis et al. (2008) reported that for whatever underlying reasons, the *mecA* gene detection and expression are prerequisites for methicillin resistance, even though such a genotype may not guarantee phenotypic methicillin resistance. However, he warned that precautions should be taken when treating OS-MRSA strains with beta-lactam antibiotics as this may result in the emergence of highly resistant MRSA, which is attributable to the presence of the *mecA* gene**

**Based on this, many researchers (Shittu et al., 2011; Thapaliya et al., 2017; Dalman et al., 2019), including us in this paper, reported and are still reporting MRSA based on the detection of the *mecA* gene**

8. Line 211: tell the readers why you used the spa gene! Not everyone is a Staph person. Same for the significance of the repeat successions.

**Response: We have added an explanation of what the spa gene is and the importance of the repeat successions in the introduction**

9. Line 214: what is the significance of saying they were “automatically” submitted?

**Response: This is merely to note they have been added to the database. This has been edited slightly.**

10. Finally on line 221 they define CC. They do list a bunch of t numbers but they have not adequately defined the importance of t numbers. I work in a different species of Staph and I don’t know what the importance of t numbers, or spa types. Thus, the impact factor of the findings is minimized. How does ANY of this relate to the global picture of S. aureus? Place Nigeria in the context of the S. aureus pan genome.

**Response: This is done in the discussion section, where we believe it is more appropriate. However, the spa types (t) and clonal complexes (CC) have been given prior explanation and definition in the introduction section. We have also related the spa types we got to the global picture of S. aureus, placing Nigeria in the context of the S. aureus pan genome**

11. BURP is used many times before it is defined in the title to Figure 1. Figure 1 is very uninformative and poorly introduced. Evidently CC084 and t084 are related some how? What is the significance of the size of the circles or the colors?

**Response: This has been edited. BURP has been defined and explained in the materials and methods.**

Figure 1 is very uninformative and poorly introduced-

**Response: This has been edited**

The significance of the size of the circle is already noted in the text: “**** Each node represents a *spa* type. The size of a node represents the number of isolates assigned to that *spa* type”. We have also explained the significance of the colours**

12. I am perplexed why the spa typing was not related to the drug resistance profiles. That seems to be a serious under-utilization of what they have set up. They could relate spa profiles with drug resistance but they ignore it and only focus on the host source.

**Response: This has been done**

13. I know there is still pervasive antibiotic use in Nigeria but that is not mentioned. Was there any survey of antibiotic administration in the flocks/herds they sampled or are they all on AGP (antibiotic growth promoters)?

**Response: This was mentioned in Lines 309-402 and 405-406. Nevertheless, we added more explanations**

14. Finally at line 356 they start explaining the relevance of spa typing and BURP. But after they used it.

**Response: We believe a discussion of this is most appropriate in this current location. However, we explained the relevance of spa typing in the introduction and BURP in the materials and methods, so that this would be most appropriate here**

15. Line 361 they start to bring in a more global perspective but then dodge away.

**Response: We have added more global perspectives**

16. They intimated that they did MLST but they did not. they used single locus typing. I actually had to go to the Ridom server and find the Home page to figure that out.

**Response: We have modified it**

Overall: the results are worth publishing but not in the form presented. They need to go back and reanalyze their data without solely focusing on Nigeria. There is a big literature about clades of S. aureus out there. They could place the Nigeria samples in that context, without making it all just about Nigeria. They can re-work this paper and make it more readable for a wider audience and focus on the need to understand the population structure of S. aureus clades in Nigeria domestic animals and the handlers. It is not at all surprising that they can isolate pig or chicken isolates from handlers. There are numerous examples of that, but are any of them causing disease? See the PNAS paper by Ross Fitzgerald many years ago for an example.

**Response: We thank the reviewer for this comment. However, we think working to fill in the data gap in developing countries including Nigeria is a critical aspect of understanding the global epidemiology of this pathogen, and is the niche and audience we are aiming for in this publication.**

**This study was not designed to analyze disease, but carriage/colonization/contamination of carcasses. We agree disease is an important aspect but is much more difficult to examine even in wealthy countries (see eg Smith & Wardyn review, Human Infections with Staphylococcus aureus CC398). We agree this would be ideally examined in future studies.**

Reviewer #2: This is a report of a cross-sectional study of S. aureus contamination of 600 chicken and 600 pig carcasses from slaughterhouses in Nigeria. There was an appropriate sampling strategy and the swabs were taken from the surface of the carcass. The chicken carcasses were handled differently (dipped in a communal water bath) than the pig carcasses which could explain the higher prevalence of contamination. In addition, they report on anterior nares colonization of 45 workers with chicken carcass contact and 45 workers with pig carcass contact. The prevalence of contamination was 4.7% in chicken carcasses, 2.8% in pig carcasses. Prevalence of anterior nares colonization was 11.0% and 6.7% respectively among the worker which is low. The low colonization rates suggests that these workers are not at increased risk because of their occupations. Spa typing, detection of the mec and PVL genes and antibiotic susceptibility was performed and analyzed appropriately. The results are presented in a very detailed fashion.

Suggestions for improving the discussions:
1. The rationale for the study is to look at animal-to-human transmission. There are overlapping spa types between workers and carcasses; however, you do not know whether the workers contaminated the carcass with SA or the carcasses transmitted SA to the workers because of the cross-sectional design. This limitation is not mentioned. There was no clear mention of what proportion of the SA isolates were livestock associated spa types; it appears from the discussion at least some were human spa types. This key point should be given more attention.

**Response: This has been added**

2. There was no discussion of how this compares to contamination with optimal slaughterhouse practices and what might change this contamination.

**Response: This has been added**

The conclusion that this is an urgent public health threat is overstated.
**Response: This has been edited**

Suggestions for improving the abstract:
1. Include the % of carcasses and workers contaminated/colonized
2. Overall #’s are distracting e.g. 1200 food animals and 90 workers- just include 600 chickens and 600 pigs and 45 chicken workers and 45 pig workers

**Response: We have modified this.**
Suggestions for Methods:
1. Include how the carcasses were swabbed – for example how much of the carcasses was sampled? How does this compare to other studies that have been done?

**Response: This has been done**
