# Supplementary material for: Molecular epidemiology, genetic diversity and antimicrobial resistance of Staphylococcus aureus isolated from chicken and pig carcasses, and carcass handlers
Source: PLoS One. 2020 May 14;15(5):e0232913. doi: 10.1371/journal.pone.0232913 (PMC7224487; doi:10.1371/journal.pone.0232913)
Supplement: Attachment — Submitted filename: Response to Reviewers.docx [file pone.0232913.s002.docx]

Reviewers' comments:

Reviewer's Responses to Questions

**Comments to the Author**

1. If the authors have adequately addressed your comments raised in a previous round of review and you feel that this manuscript is now acceptable for publication, you may indicate that here to bypass the “Comments to the Author” section, enter your conflict of interest statement in the “Confidential to Editor” section, and submit your "Accept" recommendation.

Reviewer #1: (No Response)

Reviewer #2: All comments have been addressed

2. Is the manuscript technically sound, and do the data support the conclusions?

The manuscript must describe a technically sound piece of scientific research with data that supports the conclusions. Experiments must have been conducted rigorously, with appropriate controls, replication, and sample sizes. The conclusions must be drawn appropriately based on the data presented.

Reviewer #1: Yes

Reviewer #2: Yes

3. Has the statistical analysis been performed appropriately and rigorously?

Reviewer #1: Yes

Reviewer #2: Yes

4. Have the authors made all data underlying the findings in their manuscript fully available?

The [PLOS Data policy](http://www.plosone.org/static/policies.action#sharing) requires authors to make all data underlying the findings described in their manuscript fully available without restriction, with rare exception (please refer to the Data Availability Statement in the manuscript PDF file). The data should be provided as part of the manuscript or its supporting information, or deposited to a public repository. For example, in addition to summary statistics, the data points behind means, medians and variance measures should be available. If there are restrictions on publicly sharing data—e.g. participant privacy or use of data from a third party—those must be specified.

Reviewer #1: Yes

Reviewer #2: Yes

5. Is the manuscript presented in an intelligible fashion and written in standard English?

PLOS ONE does not copyedit accepted manuscripts, so the language in submitted articles must be clear, correct, and unambiguous. Any typographical or grammatical errors should be corrected at revision, so please note any specific errors here.

Reviewer #1: No

Reviewer #2: Yes

6. Review Comments to the Author

Please use the space provided to explain your answers to the questions above. You may also include additional comments for the author, including concerns about dual publication, research ethics, or publication ethics. (Please upload your review as an attachment if it exceeds 20,000 characters)

Reviewer #1: Review PONE-D-20-02752R1
Molecular epidemiology, genetic diversity and antimicrobial resistance of Staphylococcus aureus isolated from chicken and pig carcasses and carcass handlers
Comments to authors:
1. Need a comma after carcasses in article title

**Response: It has been added.**

2. Line 31: Unnecessary to include ‘(S. aureus)’

**Response: This has been edited.**

3. Line 32: This study not The study

**Response: This has been edited.**

4. Line 34: include punctuation to properly associate the conjunctions. Perhaps they need a copy editor to clean up the punctuation and English.

**Response: This has been edited.**

5. Line 38, 46, 101: same problem. I will leave this issue to the editor to resolve, and not comment further.

**Response: This has been edited.**

6. Line 50-51: using abbreviations for antibiotics that are not defined.

**Response: This has been edited.**

7. Line 58 same problem as line 31

**Response: This has been edited.**

8. Line 130: purposively is not needed

**Response: It has been deleted.**

9. Lines 227-228: Suggest the section title as: Clustering of isolates by spa typing and BURP analyses

**Response: This has been done.**

10. Line 297 vs 305: antimicrobial resistance profile changes to antibiotic resistant profile and then they define an acronym. Suggest you choose one term and define early. Makes it cleaner.

**Response: This has been edited. We changed all to antimicrobial resistance**

11. Line 68, 201 and 307: MDR defined already

**Response: This has been edited.**

12. Line 320-321 vs 297-299: they already indicated earlier that ‘All the isolates from the four sources were resistant to penicillin’ and they seem to be repeating information in a slightly different manner in these two places. Suggest they compare these two paragraphs and remove redundancies.

**Response: This has been edited and all redundancies removed**

13. Line 330-1: what is the point and what is the basis for the beta-lactamase screening. They just seem to be repeating numbers from the tables and they would be better just pointing to where they are drawing results from. The results still seem to just be a recitation of numbers. Just trying to encourage them to consider their readers for the importance of some of the numbers.

**Response: This has been edited and since beta-lactamase was not properly discussed, we have removed it.**

14. Line 334: they are back to using Staphylococcus aureus!

**Response: This has been edited.**

15. Line 366: don’t need to redefine MSSA and MRSA.

**Response: This has been edited.**

16. The bibliography needs cleaning up, to meet PLoS One format. Only some journal titles are abbreviated. I am certain the editors will catch this on acceptance.

**Response: This has been edited too.**

Overall: the manuscript is far superior and highlights the importance of the work, with a focus on fitting into the global and African importance of the work. They need to clean up the coverage in the results, fix some punctuation, and get it published.

**Response: We thank reviewer 1 for his thorough revisions, and all have been addressed in the revised manuscript**

Reviewer #2: This is a report of a cross-sectional study of S. aureus contamination of 600 chicken and 600 pig carcasses from slaughterhouses in Nigeria. There was an appropriate sampling strategy and the swabs were taken from the surface of the carcass. The chicken carcasses were handled differently (dipped in a communal water bath) than the pig carcasses which could explain the higher prevalence of contamination. In addition, they report on anterior nares colonization of 45 workers with chicken carcass contact and 45 workers with pig carcass contact. The prevalence of contamination was 4.7% in chicken carcasses, 2.8% in pig carcasses. Prevalence of anterior nares colonization was 11.0% and 6.7% respectively among the worker which is low. The low colonization rates suggests that these workers are not at increased risk because of their occupations. Spa typing, detection of the mec and PVL genes and antibiotic susceptibility was performed and analyzed appropriately. The results are presented in a very detailed fashion.

All of my prior suggestions have been incorporated.

**Response: We also thank reviewer 2 for his comments.**
